# Endoscopic Trans Planum Trans Inter Cavernous Sinus (ETPTICS) approach to pituitary microadenoma: introduction of a novel technique to enhance endocrinological outcome

**Published:** 2012-11

**Authors:** Guive Sharifi, Maryam Jalessi, Amin Jahanbakhshi, Misagh Shafizad

**Affiliations:** ^a^Neurosurgery Department, Loghman Hakim Hospital, Shaheed Beheshti University of Medical Sciences, Tehran, Iran.; ^*b*^Endoscopic Pituitary and Skull Base Surgery Center, ENT-Head & Neck Research Center and Department, Hazrat Rasoul Akram Hospital, Tehran University of Medical Sciences, Tehran, Iran.

## Abstract

**Background:**

Endoscopic transnasal approach to pituitary adenoma surgery has been recently introduced as an efficient alternative for traditional transcranial and conventional microscopic transnasal approaches. Recent studies have shown the advantages of this technique such as better cosmetic outcomes, low complication rate, lower duration of the surgery, higher patient satisfaction, and better tumor resection, compared to microscopic technique especially in large tumors with extensions into adjacent structures and tissues. Surgery for secreting microadenoma has been always a big surgical challenge in term of achieving full treatment especially in Cushing’s syndrome.

**Methods:**

A total of 12 cases secretory microadenoma was performed during 2011. Transplanum trans-intercavernous sinus approaches were used for better searching of the gland for microadenoma and checking the dural side adjacent for microadenoma since they were very useful in multi microadenoma and bilateral cases detections. In this approach, conventional techniques step by step were followed until the sellar floor was perforated. At this step, more resection of sellar bone was performed both anteriorly and posteriorly toward planum sphenoidale to expose intercavernous sinuses. Then, following careful bipolar cautery of the intercavernous sinuses, they were incised along with dura of the sellar floor. This approach makes an excellent view of the sella and pituitary adenoma as well as the pituitary gland itself and its stalk.

**Results:**

A total of 330 cases of pituitary adenoma underwent the endoscopic transsphenoidal (ETSS) surgery during 2005-2012. Of them, there were 27 cases of microadenoma, 15 of which underwent the surgery through classic ETSS and the rest (12 patients) were operated using ETPTICS. In the first group (conventional ETSS), 11 patients (73%) were completely recovered and only 2 cases showed recurrence after one year (both were Cushing’s syndrome).

In the second group (ETPTICS), postoperative follow-up (5 months) assessments showed that all of 12 patients showed remission criteria with no recurrence. There was no postoperative cerebrospinal fluid (CSF) leak or any complication in both groups. There were 2 cases of transient diabetes insipidus (DI, unrelated to diabetes mellitus) in the ETSS group, while the other group showed 4 cases. However no permanent DI was observed in both groups.

**Conclusions:**

Findings of the present study showed that application of ETPTICS approach can increase the endocrinological remission rate that was a formidable task in cases with multiple microadenoma or Cushing’s syndrome with normal MRI and microadenoma touching the cavernous sinus or invading the dura.

Hypothetically, the chance of CSF leak might be higher in this novel technique that was efficiently controlled using meticulous repair. Furthermore, meticulous care by pituitary stalk blood supply should be implemented to avoid anterior pituitary insufficiency and permanent DI. Tin tituitary insufficiency and permanent DI are to be avoided.

**Keywords:**

Endoscopic Transplanum Tran intercavernous sinus (ETPTICS), Pituitary, Endocrinological


					Volume 4, Suppl. 1 Nov 2012
Publisher: Kermanshah University of Medical Sciences
URL: http://www.jivresearch.org
Copyright:
Congress of Iranian Neurosurgeons, 14-16 November 2012, Kermanshah, Iran
Paper No. 38
Endoscopic Trans Planum Trans Inter Cavernous Sinus
(ETPTICS) approach to pituitary microadenoma: introduction
of a novel technique to enhance endocrinological outcome
Guive Sharifi a
, Maryam Jalessi b
, Amin Jahanbakhshi a
, Misagh Shafizad a,*
a
Neurosurgery Department, Loghman Hakim Hospital, Shaheed Beheshti University of Medical Sciences, Tehran, Iran.
b
Endoscopic Pituitary and Skull Base Surgery Center, ENT-Head & Neck Research Center and Department, Hazrat Rasoul
Akram Hospital, Tehran University of Medical Sciences, Tehran, Iran.
Abstract:
Background: Endoscopic transnasal approach to pituitary adenoma surgery has been recently introduced as an efficient
alternative for traditional transcranial and conventional microscopic transnasal approaches. Recent studies have shown the
advantages of this technique such as better cosmetic outcomes, low complication rate, lower duration of the surgery, higher
patient satisfaction, and better tumor resection, compared to microscopic technique especially in large tumors with
extensions into adjacent structures and tissues. Surgery for secreting microadenoma has been always a big surgical
challenge in term of achieving full treatment especially in Cushing's syndrome.
Methods: A total of 12 cases secretory microadenoma was performed during 2011. Transplanum trans-intercavernous sinus
approaches were used for better searching of the gland for microadenoma and checking the dural side adjacent for
microadenoma since they were very useful in multi microadenoma and bilateral cases detections. In this approach,
conventional techniques step by step were followed until the sellar floor was perforated. At this step, more resection of
sellar bone was performed both anteriorly and posteriorly toward planum sphenoidale to expose intercavernous sinuses.
Then, following careful bipolar cautery of the intercavernous sinuses, they were incised along with dura of the sellar floor.
This approach makes an excellent view of the sella and pituitary adenoma as well as the pituitary gland itself and its stalk.
Results: A total of 330 cases of pituitary adenoma underwent the endoscopic transsphenoidal (ETSS) surgery during
2005-2012. Of them, there were 27 cases of microadenoma, 15 of which underwent the surgery through classic ETSS and
the rest (12 patients) were operated using ETPTICS. In the first group (conventional ETSS), 11 patients (73%) were
completely recovered and only 2 cases showed recurrence after one year (both were Cushing's syndrome).
In the second group (ETPTICS), postoperative follow-up (5 months) assessments showed that all of 12 patients showed
remission criteria with no recurrence. There was no postoperative cerebrospinal fluid (CSF) leak or any complication in both
groups. There were 2 cases of transient diabetes insipidus (DI, unrelated to diabetes mellitus) in the ETSS group, while the
other group showed 4 cases. However no permanent DI was observed in both groups.
Conclusions: Findings of the present study showed that application of ETPTICS approach can increase the endocrinological
remission rate that was a formidable task in cases with multiple microadenoma or Cushing's syndrome with normal MRI and
microadenoma touching the cavernous sinus or invading the dura.
Hypothetically, the chance of CSF leak might be higher in this novel technique that was efficiently controlled using
meticulous repair. Furthermore, meticulous care by pituitary stalk blood supply should be implemented to avoid anterior
pituitary insufficiency and permanent DI. Tin tituitary insufficiency and permanent DI are to be avoided.
Keywords:
Endoscopic Transplanum Tran intercavernous sinus (ETPTICS), Pituitary, Endocrinological
* Corresponding Author at:
Misagh Shafizad: Neurosurgery Department, Loghman Hakim Hospital, Shaheed Beheshti University of Medical Sciences, Tehran, Iran.
Tel: 09111540432, Email: mishafizad@gmail.com, (Shafizad M.).

				

